# Protective effect of proteins extracted from *Plumeria pudica* latex on ethanol-induced gastric injury in mice

**DOI:** 10.1590/acb404025

**Published:** 2025-06-06

**Authors:** Lucas Arruda Moita, Bruna da Silva Souza, Naylla Veras de Moraes Oliveira, Ana Clara Silva Sales, Lucas Eduardo Silva Oliveira, Ana Patrícia de Oliveira, Francisca Beatriz Melo Sousa, Jand-Venes Rolim Medeiros, Jefferson Soares de Oliveira

**Affiliations:** 1 Universidade Federal do Delta do Parnaíba – Laboratório de Bioquímica de Plantas Laticíferas – Parnaíba (PI), Brazil.; 2 Universidade Federal do Delta do Parnaíba – Laboratório de Inflamação e Gastroenterologia Translacional – Parnaíba (PI), Brazil.

**Keywords:** Latex, Proteins, Stomach Ulcer, Oxidative Stress

## Abstract

**Purpose:**

To evaluate proteins from *Plumeria pudica* latex (LPPp) for their protective effect against ethanol-induced gastric injury in mice.

**Methods:**

The LPPp fraction was obtained by collecting *P. pudica* latex in tubes containing distilled water, followed by centrifugation and dialysis. The female Swiss mice (*Mus musculus*) received saline or LPPp (40 mg/kg) intraperitoneally 1 hour before oral administration of 500 μL of 50% ethanol. One hour later, the animals were euthanized, and their stomachs were removed for evaluation of tissue lesion area, histopathological analysis, and measurements of malondialdehyde (MDA), glutathione (GSH), superoxide dismutase (SOD), and nitrate/nitrite (NO_3_/NO_2_). An independent experiment assessed the effect of LPPp on gastric mucus production.

**Results:**

The LPPp-treated animals showed a significant reduction in the mean injured areas of gastric tissue (0.73 ± 1.01 mm^2^) compared to the ethanol group (37.99 ± 3.11 mm^2^). Histopathological analysis revealed significant preservation of tissue architecture in the LPPp group compared to ethanol group. Additionally, LPPp maintained tissue levels of MDA, GSH, SOD, and NO_3_/NO_2_ comparable to the saline group and influenced gastric mucus production favorably (*p* < 0.05).

**Conclusion:**

These results suggested that LPPp has a protective effect against ethanol-induced gastric lesions, likely involving antioxidant activity and increased gastric mucus production.

## Introduction

Gastric ulcer, also known as peptic ulcer, is a common digestive disease that has become a global problem, affecting 5–10% of the world’s population resulting in the expenditure of millions of dollars on healthcare^1^^–^ 3. Under normal circumstances, to protect the integrity and functionality of the gastric mucosa, the gastric mucosal barrier uses the mucus–bicarbonate–phospholipid barrier, the epithelial barrier, and the endothelial barrier^4^^,^ 5. The gastric ulcer is a complex and multi-factorial process, commonly produced by an imbalance between gastric mucosal protective and aggressive factors such as gastric acid, pepsin, reactive oxygen species (ROS) and *Helicobacter pylori* 6.

Alcohol is one of the most abused substances around in the world, which can cause upper gastrointestinal bleeding and peptic ulcers^2^^,^ 5. The excessive alcohol consumption is considered the main cause of gastric mucosal injury^6^. Excessive consumption of some alcoholic drinks is associated to human gastric damage, and the degree of injury is related to ethanol concentration and quantity^7^.

Pathogenesis of gastric ulcer includes the production of oxygen-derived free radicals such as superoxide anion radical, hydroxyl radicals, and lipid peroxides^8^^–^ 10. Thus, when the gastric mucosa is damaged, an unbalanced in pro-inflammatory cytokines is started, followed by neutrophils migration to the damaged site, and the concentration of ROS and other inflammatory mediators is increased, promoting an oxidative damage^11^. Additionally, ethanol changes mucosal permeability to gastric acid by causing mast cells, macrophages, and blood cells to release vasoactive products^12^. Alcohol also usually induces damage to the gastric mucosa promoting edema, erosion, ulcerative lesions, hemorrhage, and infiltration of inflammatory cells^13^.

Therefore, anti-oxidation and anti-inflammation activity seem to play an important role in protection of the gastric mucosa against damage^7^. At present available therapies do not provide definite cure of gastric ulcer. Thus, some alternative treatments are necessary^12^. Some studies have demonstrated the gastroprotective potential of some plants and its natural active constituents against experimental gastric injury in animal models^14^. Herbal medicines have been used as medicinal treatments that contain different gastroprotective mechanisms, including stimulation of mucosal proliferation, inhibition of acid production, and antioxidant properties^15^.

*Plumeria pudica* (Jacq., 1760) is an ornamental plant commonly known as bridal bouquet. It belongs to the Apocynaceae family, and is characterized by intense latex production^16^^–^ 18. A well-defined and rubber free protein fraction (LPPp) obtained from its latex previous demonstrated oxidative stress and inflammatory properties in different experimental models^19^^–^ 22, without promoting signs of toxicity in animals at therapeutic doses^23^.

The effect of latex proteins from *P. pudica* on ethanol-induced acute gastric mucosal damage has not been studied. Thus, this study evaluated the effect of this protein fraction on the acute gastric injury induced by ethanol.

## Methods

### Latex collection extraction of latex proteins

Plants of *P. pudica* from Parnaíba (PI), Brazil (2.9055°S, 41.7734°W), were used as the source of fresh latex. The plant material was identified and the voucher No. 2,432 was deposited in the Herbário Delta do Parnaíba from Universidade Federal do Piauí. The latex was collected in distilled water (1:1; v/v) and centrifuged at 3,600 × g for 15 min at 25°C. The supernatant was dialyzed against distilled water using membranes of 8 kDa. The dialysis water was renewed every six hours and centrifuged again using the conditions described above. The supernatant was lyophilized and named latex proteins from *P. pudica* (LPPp) and used for further experiments.

### Animals

Female Swiss mice (*Mus musculus*) weighing 25–30 g were supplied by the Universidade Federal do Piauí. Animals were housed in cages with free access to food and water and were maintained under a 12-h light–dark cycle (lights on at 6 a.m.) at 24 ± 2°C. All experimental procedures were performed as specified by the Guide for Care and Use of Laboratory Animals (National Institute of Health, Bethesda, MD, United States of America), and Institutional Animal Ethics Committee from Universidade Federal do Piauí, that approved the project (Protocol No. 470/18).

### Ethanol-induced gastric damage

The experiments were conducted following the method described by Robert et al.^24^, with some modifications. The mice were divided into three groups (5–8 animals):

- Saline group (SAL): negative control;
- Ethanol 50% group: positive control;
- Experimental group, treated with LPPp at 40 mg/kg.

The dose of 40 mg/kg was chosen since it was the best dose that LPPp showed anti-inflammatory and antioxidant activity in other *in-vivo* experimental models^19^^–^ 22, and it was the dose recommended by Institutional Animal Ethics Committee to perform our evaluation.

Before the experiment, the animals were starved of food for 15 hours and water for 2 hours. After this period, gastric lesion was induced by single administration of 0.5 mL/25 g of 50% ethanol by gavage. The negative control group received an equivalent volume of saline solution (0.9%). Animals were treated with LPPp (40 mg/kg, intraperitoneally) 1 hour before ethanol administration. One hour after administration of ethanol, all animals were euthanized, their stomachs were immediately removed, opened via an incision along the greater curvature and pinned out on a wax block. Gastric damage was measured using a computer planimetry program (Image J). A sample of the corpus region of each stomach was fixed in 10% formalin immediately after removal for subsequent histopathological assessment. Further, gastric corpus samples were frozen and stored at -80°C for biochemical analysis.

### Histopathological analysis

For histopathological evaluation, corpus region of each stomach fixed in 10% formalin solution were sectioned and embedded in paraffin. Four-micrometer-thick sections were deparaffinized, stained with hematoxylin and eosin, and then examined under a microscope. Samples were then analyzed in a blind study (without knowledge of the previous treatments) by an experienced pathologist using procedure adapted25. Briefly, it was examined 1-cm-long sections for epithelial cell loss (a score of 0–3), presence of inflammatory cells (a score of 0–3), edema in the upper mucosa (a score of 0–4), and haemorrhagic lesion (a score of 0–4).

### Determination of adhered mucus to gastric wall

The mucus content determination was performed as described by Corne et al.26 after ethanol induced gastric ulcer. Segment of the glandular region of the stomach was weighted and transferred to a test tube containing 3 mL of 0.1% Alcian blue for 2 hours. After two consecutive rinses with 3 mL of sucrose (0.25 M), 3 mL of MgCl2 (0.5 M) was added in each test tube. The glandular segment remained in this solution for 2 h, with intermittent agitation. Afterwards, 4 mL of the resultant blue solution was agitated vigorously with 4 mL of ethyl ether until the formation of an emulsion and centrifuged at 3,600 × g for 10 min. The absorbance of the supernatant was read at 598 nm. The concentration of Alcian blue was expressed in µg Alcian blue/g of glandular tissue.

### Glutathione concentration

The concentration of reduced glutathione (GSH) in stomach tissues as nonprotein sulfhydryl was estimated using the technique described by Sedlak and Lindsay27. Results were expressed as µg of GSH/g of tissue.

### Malondialdehyde levels

The level of malondialdehyde (MDA) in stomach homogenate samples was measured using the method described by Mihara and Uchiyama^28^. Results were expressed as nmol of MDA/g of tissue.

### Superoxide dismutase activity

Superoxide dismutase (SOD) activity was measured using spectrophotometric assay decribed by Das et al.^29^. In addition, total proteins concentration in each homogenate sample was determined with a commercial kit from Labtest. Results were expressed as unit of SOD (USOD)/μg of protein.

### Levels of nitrate/nitrite

The level of nitric oxide (NO) was obtained by quantifying the NO metabolites nitrate (NO_3_^-^) and nitrite (NO_2_^-^) in the gastric tissue, according to the method described by Green et al.^30^. Results were expressed as μM of NO_3_/NO_2_.

### Statistical analysis

The results were expressed as mean ± standard error of mean (± SEM) of n = 5–8 animals. Differences between groups were evaluated using analysis of variance and the Student–Newman–Keuls post-test, when appropriate. Moreover, the Kruskal-Wallis nonparametric test, followed by Dunn’s test, were used in histopathological analyses. Difference between groups were considered statistically significant when *p* < 0.05. Statistical analysis was performed using GraphPad Prism statistical software, version 7.0.

## Results

## Latex proteins from Plumeria pudica reduced injured areas in stomachs

**Figure 1:**
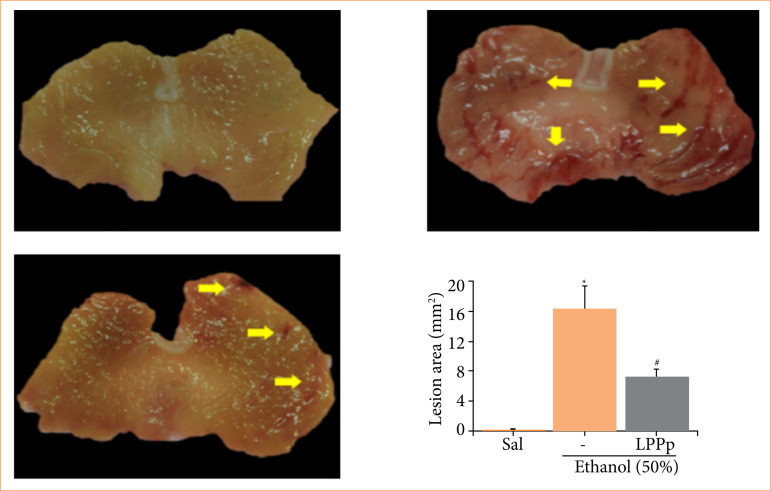
Effect of proteins extracted from *Plumeria pudica* latex (LPPp) on injured areas in stomachs of ethanol-induced gastric lesion. **(a)** Saline group. **(b)** Ethanol. **(c)** Mice were treated with LPPp (40 mg/kg) intraperitoneally. After 30 min, **(d)** the animals in experimental groups were administered 50% ethanol. **(a–c)** Images of stomachs were captured, and **(d)** lesion areas assigned in the planimetric software were expressed in mm^2^. Yellow arrows indicate the presence of lesion areas. Results are expressed as mean ± standard error of the mean of 5–8 animals per group. Pictures are representative samples of tissues from every animal.

Macroscopic analysis of the gastric mucosa of animals demonstrated that LPPp was able to protect the frontal mucosa from injuries produced by ethanol. In the ethanol group, extensive tissue lesions were observed in the form of stretch marks when compared to the saline group (Figs. 1a and b). In contrast, the animals in the LPPp group, that also received ethanol, had fewer areas of lesions (Fig. 1c). Moreover, computerized planimetric measurement of the injured areas in the stomach of all groups indicated that the animals treated with LPPp had areas of injury in the gastric wall significantly smaller (7.28 ± 1.00 mm^2^) than those of the animals belonging to the ethanol group (16.30 ± 3.11 mm2) (Fig. 1d). The effect produced by LPPp corresponded to 56% of inhibition of lesion.

*p < 0.05 versus saline group; #*p* < 0.05 versus ethanol group (analysis of variance followed by Student-Newman-Keuls test). Source: Elaborated by the authors.

## Latex proteins from Plumeria pudica inhibited histopathological alterations of gastric tissue

**Figure 2:**
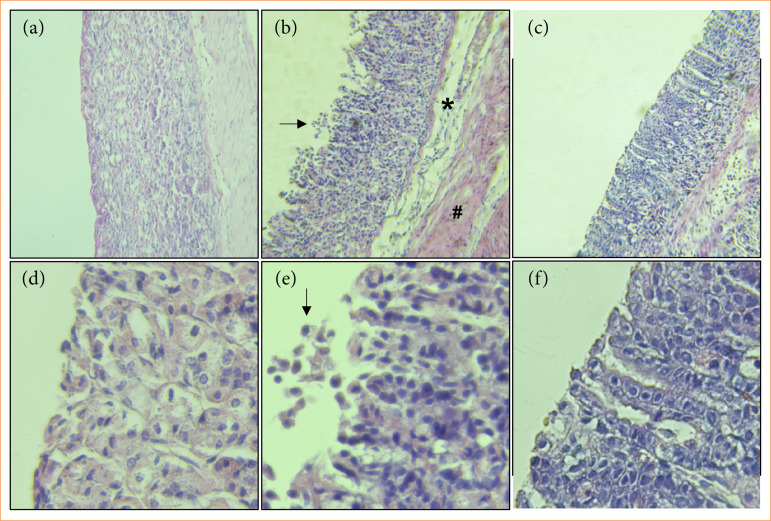
Effect of proteins extracted from *Plumeria pudica* latex (LPPp) on microscopic changes in stomachs of ethanol-induced gastric lesion. **(a, b** and **c)** Magnification of 100x. **(d, e** and **f)** Magnification of 400x. **(a** and **d)** Gastric mucosa of mice from saline group with normal architecture. **(b** and **e)** Presence of edema (*), haemorrhagic areas (#) and los of architecture (arrows) in ethanol group. **(c** and **f)** Stomach of animals treated with LPPp (40 mg/kg; intraperitoneally) which exerted a protective effect on gastric mucosa with preservation of epithelial cells and absence of haemorrhages. Pictures are representative samples of tissues from every animal. Quantitative results from these assessments are shown in Table 1.

Source: Elaborated by the authors.

Oral administration of ethanol disrupts the integrity of the gastric mucosa with excessive loss of epithelial cells, which causes a rupture in the surface of the mucosa, besides accentuating edema and hemorrhage (Fig. 2). However, administration of LPPp maintained the integrity of the mucosa, showing that LPPp exerted a potential gastroprotective effect on this lesion. The histopathological evaluation showed that LPPp significantly decreased epithelial cell loss, edema, and hemorrhagic damage induced by ethanol administration (Fig. 2c and 2f, Table 1). No significant difference was observed in inflammatory cell.

**Table 1:** Histopathological assessment of gastric mucosa of mice treated with proteins extracted from Plumeria pudica latex (LPPp). Data represent the median and range of scores.

Microscopic parameters	Score	Saline	Ethanol	LPPp
Epithelial cell loss	0–3	0 (0–1)	3 (2–3)*	1 (1–2)#
Presence of Inflammatory cells	0–3	1 (0–1)	1 (1–1)	1 (0–1)
Edema in the upper mucosa	0–4	0 (0–1)	4 (2–4)*	2 (1–2)#
Hemorrhagic damage	0–4	0 (0–1)	3 (2–4)*	1 (0–2)#
Total score	0–14	1 (2–3)	11 (9–10)*	5 (2–3)#

Data represent the median and range of scores.

* *p* < 0.05 *versus* saline group;

# *p* < 0.05 *versus* ethanol group (analysis of variance followed by Kruskal-Wallis and Dunn’s tests).

Source: Elaborated by the authors.

## Latex proteins from Plumeria pudica increased glutathione concentration

**Figure 3:**
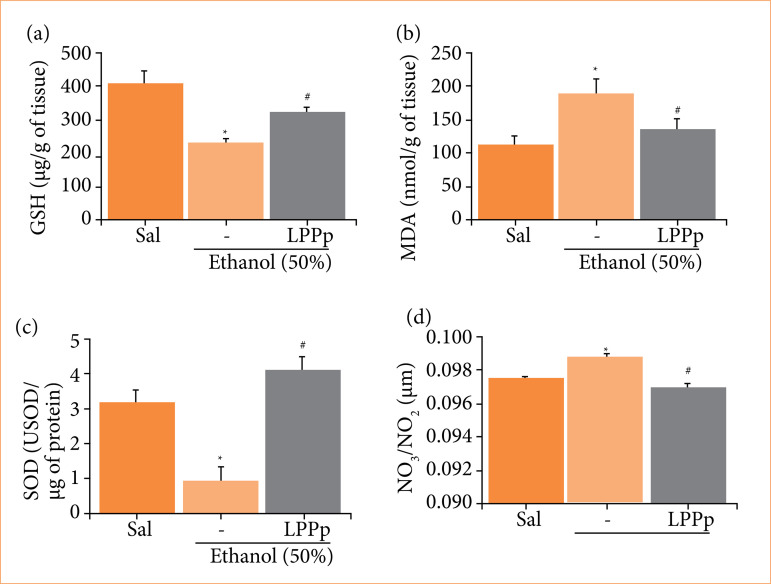
Effect of proteins extracted from *Plumeria pudica* latex (LPPp) on the levels of **(a)** glutathione (GSH), **(b)** malondialdehyde (MDA), **(c)** superoxide dismutase activity (SOD), and **(d)** total nitrate and nitrite concentration in gastric mucosa of ethanol-induced lesion. Mice were treated with LPPp (40 mg/kg) intraperitoneally. After 30 min, the animals in experimental groups were administered 50% ethanol. Results are expressed as mean ± standard error of the mean of 5–8 animals per group.

The measurement of GSH in the gastric tissue of animals indicated that animals receiving only ethanol had a significant consumption of GSH in the mucosa (231.3 ± 16.3 µg/g of tissue) compared to the saline group (406.7 ± 39.8 µg/g of tissue) (Fig. 3a). However, compared to ethanol group, pre-treatment of animals with LPPp induced an increment of GSH in the mucosa (321.8 ± 17.7 µg/g of tissue).

*p < 0.05 versus saline group; #*p* < 0.05 versus ethanol group (analysis of variance followed by Student-Newman-Keuls test). Source: Elaborated by the authors.

## Latex proteins from Plumeria pudica reduced malondialdehyde levels

Figure 3b shows the result of measurement of MDA in gastric mucosa of animals. Ethanol group presented values of MDA of 188.8 ± 22.6 nmol/g of tissue, which was significantly higher than saline group (113.5 ± 12.1 nmol/g of tissue). Animals pre-treated with LPPp showed a significant reduction in gastric MDA levels (134.8 ± 16.9 nmol/g tissue) compared to the ethanol group.

## Latex proteins from Plumeria pudica augmented superoxide dismutase activity

Analysis of the enzymatic activity of SOD in the stomachs of the animals revealed that the ethanol significantly decreased the activity of SOD in the gastric mucosa of the mice (0.94 ± 0.39 USOD/µg of protein) compared to the group that received only saline (3.16 ± 0.37 USOD/µg of protein) (Fig. 3c). On the other hand, animals treated with LPPp was able to normalize SOD activity (4.14 ± 0.35 U SOD/µg of protein), reversing the effect promoted by ethanol.

## Latex proteins from Plumeria pudica reduced NO_3_/NO_2_ concentration

Mice belonging to the group that received LPPp presented significant reduction in the levels of nitrate/nitrite in the gastric mucosa (0.0972 ± 0.0002 µM) compared to the ethanol group (0.0990 ± 0.0003 µM) (Fig. 3d). Values of NO_3_/NO_2_ in saline group were 0.0977 ± 0.0002 µM.

Latex proteins from Plumeria pudica increased adhered mucus to gastric wall

**Figure 4:**
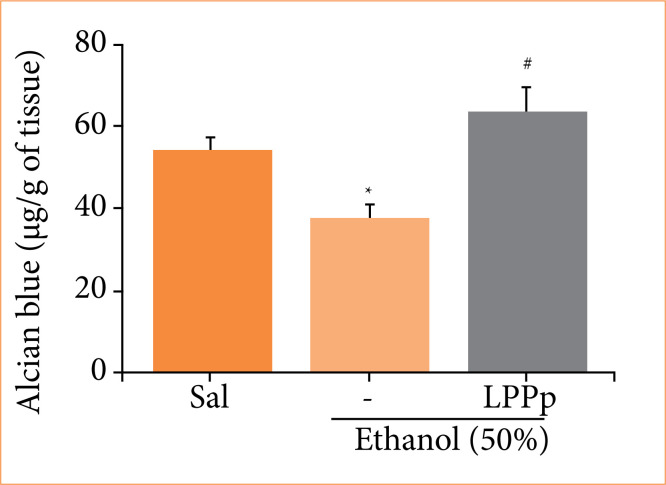
Effect of proteins extracted from *Plumeria pudica* latex (LPPp) on Alcian blue concentration in gastric mucosa of ethanol-induced lesion. Mice were treated with LPPp (40 mg/kg) intraperitoneally. After 30 min, the animals in experimental groups were administered 50% ethanol. Results are expressed as mean ± standard error of the mean of 5–8 animals per group.

Quantification of Alcian blue adhered to gastric mucosa of animals showed that ethanol group had a significant lower amount of Alcian blue (37.3 ± 3.9 µg/g of tissue) compared to the saline group (54.4 ± 3.4 µg/g tissue) (Fig. 4). However, the treatment of animals with LPPp presented higher mean of AB (64.1 ± 5.7 µg/g of tissue) in relation to ethanol group.

*p < 0.05 versus saline group; #*p* < 0.05 versus ethanol group (analysis of variance followed by Student-Newman-Keuls test). Source: Elaborated by the authors.

## Discussion

Gastric injury induced by ethanol promotes the formation of local ulcers, characterized by extensive red macroscopic lesions, hemorrhagic streaks, as well as microscopic tissue damage such as edema, loss of epithelial cells, and infiltration of inflammatory tissue^31^^,^ 32. Its fast-damaging effect occurs through the direct action on the protective mucus layer of the tissue, weakening the mucosa against the hydrolytic action of HCl and the proteolytic action of pepsin^33^. Studies have documented the anti-ulcerative properties of medicinal plants and their active compounds through animal experimentations^14^^,^ 34.

Thus, in the present study, we investigated the ability of the LPPp to protect the gastric mucosa of animals against ulcerative lesions induced by ethanol. Different studies have shown that LPPp administered in different doses by intraperitoneal route, especially at the dose of 40 mg/kg, has promising pharmacological potential, showing action in different pharmacological models. Among the activities already investigated, it should be highlighted its anti-inflammatory and antinociceptive action^19^ antidiarrheal properties^20^, protective effect against ulcerative colitis^21^, and the reduction of alveolar bone loss in periodontal disease^22^. Besides, no toxic effects were observed when LPPp is administered to animals in chronic and sub-chronic assays^23^.

In the present study, the pre-treatment of animals with LPPp significantly influenced the ulcerogenic process caused by the administration of ethanol. LPPp reduced the areas of gastric lesion, hemorrhage, and necrosis in the epithelial cells in the tissue. This effect was confirmed by histopathological evaluation, which demonstrated preservation of tissue architecture, the few losses of epithelial cells and reduction in tissue edema in animals that received LPPp. Gastroprotective activity promoted by proteins extracted from other laticifer fluids has been described in the literature^31^^,^ 35. The protein fractions from *Himatanthus drasticus* and *Plumeria rubra* also reduced the injured areas and the microscopic scores of lesions caused by oral administration of ethanol.

In addition to the observed effect of LPPp on macroscopic and microscopic changes induced by ethanol on animals’ gastric tissue, we investigated the effect of the fraction on the endogenous antioxidant system. It is known that the oral administration of ethanol causes several effects, such as intense lesions in the gastric mucosa due to the strong generation of free radicals, as well as by the imbalance between oxidative factors and antioxidant defenses^2^^,^ 33. In this context, some studies^21^^,^ 31 ^,^ 36 observed that the oral administration of ethanol promoted significant changes in several markers related to tissue oxidative stress, such as intense consumption of GSH, increased levels of MDA, depletion in the action of SOD, and increase in the concentration of NO_3_/NO_2_ in the gastric tissue of animals.

Clinical and experimental findings suggested that substances with antioxidant potential can promote gastroprotective effects^37^^,^ 38. We observed in the present study that the treatment of animals with LPPp reduced the tissue levels of MDA, as well as avoided the consumption of GSH and preserved the capacity of action of SOD, despite the gastric lesion induced by the administration of ethanol.

Oxidative stress is a noticeable feature in the damaged gastric mucosa^39^. SOD and GSH are the main antioxidant enzymes involved in the eradication of oxygen radicals and are considered an important antioxidant defense in gastric cells against ethanol-induced oxidative stress^7^. In this context, MDA and GSH are two important biochemical markers of tissue oxidative stress^33^. The increase in MDA levels is related to the occurrence of lipid peroxidation, and its level is associated to the severity of oxidative stress^32^^,^ 39. GSH participates in the oxidative process initiated by glutathione peroxidase and is fundamental for the regulation of the glutathione redox cycle^40^^,^ 41. As important free radical scavenging enzyme in the body, SOD can eliminate and neutralize ROS and free radicals, thus, to protect the gastric mucosa^2^. The decrease of SOD activity and GSH levels may also disturb the healing of gastric mucosal erosions^39^.

In this sense, our results demonstrated the antioxidant action performed by the fraction on oxidative action of reactive metabolites. The antioxidant potential of LPPp had already been observed in previous studies. It was demonstrated the efficacy of LPPp by reducing MDA levels and avoiding the consumption of GSH in the intestines of animals with diarrhea induced by castor oil^20^, in the liver of animals subjected to periodontal disease^22^, and acetic acid-induced ulcerative colitis, as well as preservation in SOD activity^21^.

In comparison to other studies involving proteins recovered from latex and their gastroprotective action on the antioxidant system, the study conducted with proteins from *H. drasticus* also observed preservation of GSH levels^35^, similar to the findings of the present study. In addition, in the evaluation performed with the protein fraction from *P. rubra*, it was also possible to observe a restoration of GSH levels in the face of the harmful effects caused by ethanol^31^.

Gastric mucosal defense is controlled by NO through blood flow regulation, mucus release, and inhibition of inflammatory infiltrates^31^^,^ 42. Also, it has been reported that NO in high concentration can accentuate the symptoms of gastrointestinal diseases due to the direct effect of cytotoxicity, vasodilation, activation of neutrophils with subsequent injury to the epithelial cells^43^. The changes found in the levels of NO_3_/NO_2_ in the ethanol group corroborated findings already described in the literature^35^^,^ 42. On the other hand, in the present study, we found that there was a significant reduction in the levels of NO_3_/NO_2_ in animals pretreated with LPPp, thus suggesting that the proteins from *P. pudica* latex may also participate in the inhibition of the production of reactive species of nitrogen in the stomachs of animal. These results were expected since animals treated with LPPp also had their levels of tissue nitrites and nitrates significantly reduced during experimental colitis^21^.

In addition to the role of LPPp on antioxidant defense systems, it is likely that the observed gastroprotective effect should be related to combined actions on other tissue protection mechanisms, such as the protective mucus layer. An increase in mucus production acts as a barrier against hydrogen ion diffusion and improves the buffering effects of gastric juices, thus inhibiting gastric ulcer formation^33^. It is well described in the literature that agents that stimulate mucus production significantly reduce the risk of gastric developing lesions^43^. In the present study, an assessment was made of the physiological action of LPPp on the mucus-reducing effect caused by the administration of ethanol. As a result, it was possible to observe that LPPp was effective on gastric mucus levels when compared to the ethanol control group, thus suggesting that the protein fraction from *P. pudica* may act on factors that stimulate mucus production.

The protein fraction of *P. pudica* latex has been targeted of biochemical studies that aimed to characterize and identify its proteins. LPPp comprises proteinase inhibitors, chitinases and proteolytic enzymes, including cysteine proteinases^20^^,^ 21. As for the identification of molecules involved in the gastroprotective process, reports involving latex proteins are rare. However, a study developed by Mello et al.^44^ suggested that cysteine proteases from the latex of *Carica candamarcensis* are involved in the process of protecting the mucosa of animals against the formation of ulcers. More studies are needed to clarify the putative protein(s) present in PLPp involved with its protective effect on ethanol-induced gastric injury in mice. Taking this into consideration, we have recently started chromatographic strategies that aim to fractionate and purify proteins. The main idea is to advance on the identification of the protein(s) involved in the biological events investigated and advance on the mechanisms of action of LPPp.

## Conclusion

The present study demonstrated that PLPp treatment has significant protective effect in the model of gastric mucosal injury induced by ethanol. The effect of LPPp can be attributed to different mechanism of tissue protection, including antioxidant system and the production of gastric mucus. These experimental findings suggested LPPp as a promising candidate for treatment of gastric ulcer. Additional studies are needed to estimate the mechanism of LPPp for gastric mucosal damage.

## Data Availability

The data will be available upon request.
